# Neuromyelitis Optica: A Deceiving Disorder of Demyelination

**DOI:** 10.7759/cureus.65123

**Published:** 2024-07-22

**Authors:** Micah Pippin, Saad Hanan, Danielle Pawlichuk, Dana Mays

**Affiliations:** 1 Family Medicine, LSUHS (Louisiana State University Health Sciences) Shreveport/Rapides Regional, Alexandria, USA

**Keywords:** myelitis, acute optic neuritis, aquaporin-4 antibody, aquaporin-4, neuromyelitis optica spectrum disorder

## Abstract

Neuromyelitis optica (NMO), also known as Devic syndrome, is an autoimmune inflammatory and demyelinating disorder that affects the optic nerves and spinal cord. It is believed to be attributed to aquaporin-4 antibodies, a water channel expressed on astrocytes. It commonly presents with isolated or recurrent attacks of myelitis and optic neuritis. Intractable vomiting and hiccups may also be seen as symptoms. Acute treatment is often achieved with high-dose steroids and is imperative to prevent permanent central nervous system damage. Relapse prevention is achieved using long-term immunosuppression. This paper examines the case of an African-American female who presented with ascending lower extremity weakness.

## Introduction

Neuromyelitis optica (NMO) is an autoimmune demyelinating disorder that commonly presents with attacks of transverse myelitis and optic neuritis [1]. Previously thought to be a variety of multiple sclerosis, the recent discovery of aquaporin-4 (AQP4) water channels allowed for NMO to be distinguished as its own disease process [2]. These aquaporin channels are highly expressed throughout the central nervous system and are the most predominant water channels in the brain [2]. When the antibodies bind to the aquaporin channels, it causes a complement and cell-mediated reaction that damages astrocytes and leads to subsequent demyelination [2]. Repeated astrocyte and oligodendrocyte damage can have cumulative systemic effects.

## Case presentation

A 61-year-old African-American female with a past medical history of hypertension, hyperlipidemia, anemia, and peripheral neuropathy presented to the clinic with complaints of left lower extremity weakness and numbness for three days. Upon further examination, the patient was found to have a limited range of motion on her left side, with a notable difference in grip strength in her left upper extremity compared to her right. She spoke in complete sentences without other focal neurological deficits in speech or language.

Of note, the patient endorsed a similar episode affecting both legs several years ago. At that time, she was diagnosed with a "neurological condition," but she was unable to recall any further details. Her symptoms resolved, and she made a full recovery. She did not maintain follow-up with a neurologist as recommended. Due to the patient's reported medical history and neurological deficits noted on the physical examination, the patient was transferred to the emergency room for further evaluation.

The patient was alert and oriented in the emergency room but reported difficulty lifting her left leg. She stated she could push down with her foot but was unable to lift up.

On a physical examination, the patient was found to have focal weakness with gait abnormalities and numbness of the lower extremities, left (L) more than right (R), with strength of +1 (B/L) on leg extension and leg flexion -2 (L), -1 (R). Reflexes of the lower extremities S1/S2 were diminished on the left more than on the right. The patient's neurological exam was otherwise unremarkable, with no loss of urinary control function.

**Figure 1:**
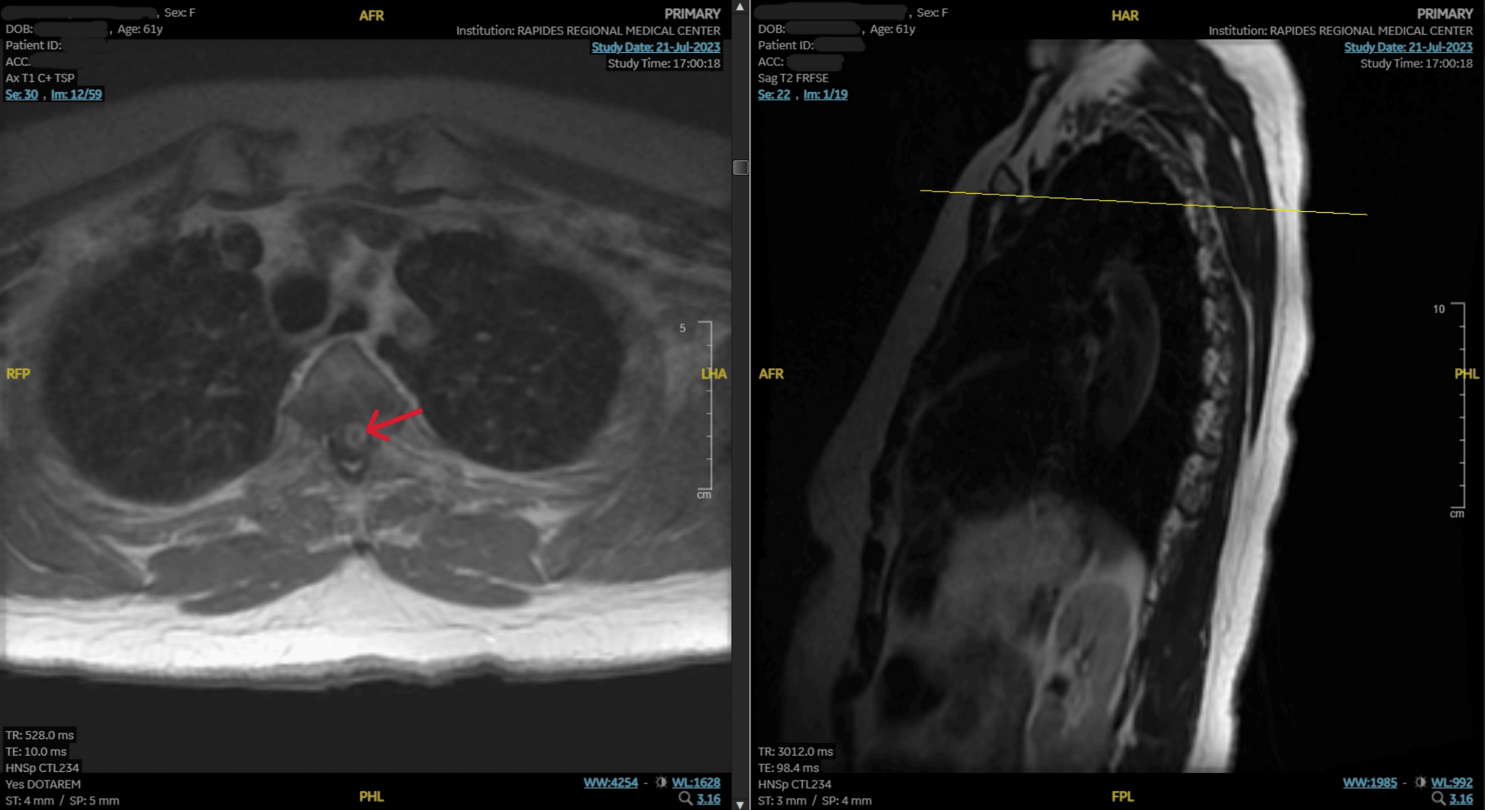
MRI demonstrating a new enhancing lesion in the upper thoracic cord MRI, magnetic resonance imaging.

Magnetic resonance imaging (MRI) of the cervical and thoracic spine with and without intravenous (IV) contrast was significant for a large new enhancing lesion in the upper thoracic cord spanning four thoracic segments (T1 to T4) with surrounding edema (Figure 1). 

The patient was started on methylprednisolone 1 g IV daily for three days per neurology, and nephrology was consulted for five days of plasma exchange. The patient received daily occupational and physical therapy throughout her hospital stay, with gradual improvement in symptoms. She was subsequently discharged to an outpatient rehabilitation facility for additional therapy. The patient was also to continue on oral prednisone 40 mg and azathioprine 100 mg daily for continued immunosuppression until outpatient follow-up with neurology.

## Discussion

NMO is a relatively rare disease that occurs across all geographic locations and ethnicities. However, higher prevalence and incidence rates have been reported in Black, Asian, and Hispanic individuals, females, and those with concomitant autoimmune conditions [2-4]. NMO can present at any age but is more commonly seen in people between the ages of 30 and 45 [3,5].

The majority of cases are caused by an auto-antibody response to AQP4 water channels in the central nervous system that leads to the destruction of astrocytes [1,3]. Depending on disease severity, there can also be secondary loss of neurons and oligodendrocytes [1,3]. More severe cases are often seen in patients with higher levels of AQP4 channel expression and subsequent antibodies [3]. However, there have been instances of NMO in which patients were seronegative for AQP4 antibodies.

Acute attacks often present with optic neuritis and transverse myelitis, plus or minus intractable nausea and hiccups [2,3]. Visual disturbances are variable but typically present with hazy vision, scotomas, and a decrease in high-contrast visual acuity [3]. Symptoms of transverse myelitis also vary based on severity, ranging from mild motor and sensory disturbances to ascending paresis and bowel/bladder incontinence [3].

Due to variability in presentation and the recent discovery of AQP4 antibodies, specific diagnostic criteria remain an ongoing and evolving discussion. The diagnosis is primarily clinical, with additional consideration given to lab tests and neuroimaging studies [1]. The presence of AQP4 antibodies is a core diagnostic indicator with 75% sensitivity and 99% specificity for NMO and is critical for differentiation from multiple sclerosis [1]. Ongoing research into NMO, its disease process, and its variants will likely lead to continuing revisions to the diagnostic criteria [4].

Treatment of NMO is divided into two categories: acute and preventative. Management of acute attacks is aimed at suppressing the initial inflammatory response with high-dose IV steroids [6]. The initial dosing is then followed by an oral steroid taper for several weeks based on the severity of the episode [6]. If improvement is not seen within the first few days of high-dose steroids, then plasma exchange with intravenous immunoglobulin (IVIG) is recommended [2,6]. Return of function after an acute attack is attributed to a reduction in spinal cord inflammation at the level of the lesion [6]. Further recovery over subsequent months is due to partial remyelination and compensatory pathways created and strengthened through ongoing therapy [6].

Prevention of future attacks is achieved through long-term immunosuppression, often with azathioprine or mycophenolate, combined with low-dose corticosteroids [2,6]. Clinical trials are currently examining the utility of biologics and disease-modifying drugs as potential agents for additional prevention of relapses [6,7]. If untreated, repeated attacks of NMO can have cumulative and detrimental neurologic effects that can lead to permanent motor deficits, blindness, and even death [2].

## Conclusions

Previously thought to fall within the realm of multiple sclerosis, recent investigation into the pathophysiology of NMO has distinguished it as its own autoimmune disease process with specific diagnostic criteria, including history, physical examination, imaging, and laboratory analysis. Prompt diagnosis, timely intervention, and subsequent therapy are imperative for optimal chances of a return to baseline neurologic function. However, delays in diagnosis or treatment, or lack thereof, can lead to cumulative neurological deficits and permanent debilitation.
